# CCR3 antagonist impairs estradiol-induced eosinophil migration to the uterus in ovariectomized mice

**DOI:** 10.1590/1414-431X20198659

**Published:** 2019-12-20

**Authors:** J.M.D. Araújo, L.A.S. Silva, F.B. Felix, E.A. Camargo, R. Grespan

**Affiliations:** 1 Laboratório de Migração Celular, Departamento de Fisiologia, Universidade Federal de Sergipe, São Cristóvão, SE, Brasil; 2 Laboratório de Processo Inflamatório, Departamento de Fisiologia, Universidade Federal de Sergipe, São Cristóvão, SE, Brasil

**Keywords:** Estradiol, Chemokine receptor, Immunology, Menopause, Uterus

## Abstract

Eosinophils are abundant in the reproductive tract, contributing to the remodeling and successful implantation of the embryo. However, the mechanisms by which eosinophils migrate into the uterus and their relationship to edema are still not entirely clear, since there are a variety of chemotactic factors that can cause migration of these cells. Therefore, to evaluate the role of CCR3 in eosinophil migration, ovariectomized C57BL/6 mice were treated with CCR3 antagonist SB 328437 and 17β-estradiol. The hypothesis that the CCR3 receptor plays an important role in eosinophil migration to the mouse uterus was confirmed, because we observed reduction in eosinophil peroxidase activity in these antagonist-treated uteruses. The antagonist also influenced uterine hypertrophy, inhibiting edema formation. Finally, histological analysis of the orcein-stained uteruses showed that the antagonist reduced eosinophil migration together with edema. These data showed that the CCR3 receptor is an important target for studies that seek to clarify the functions of these cells in uterine physiology.

## Introduction

The participation of eosinophils in the female reproductive tract has been reported since the 1960s (1). The first studies observed eosinophil migration to the uterus elicited by estrogen but not progesterone (2). In immature rats, this migration has been accompanied by uterine edema (3). Numerous studies have been conducted using different animal strains and doses and times of estrogen to promote these phenomena (2,3).

During the reproductive cycle, estrogen circulation promotes the increase in uterine eosinophils, and these cells are linked to tissue remodeling and maintenance of the normal estrous cycle in rodents (4,5). In the stages of proestrus and estrus, the rodents are susceptible to mating. Thus, eosinophils infiltrate and their proximity to the epithelial surface increases when exposed to semen (6). However, eosinophils present in the endometrium during genital pathogen infection secrete IL-4 to promote vascular stromal cell proliferation and repair of injured endometrial tissue (7).

The relationship of endocrine mediators, particularly estrogen, in regulating cell development and pathways in the innate and adaptive immune system is well documented (8). Furthermore, another study showed that steroid hormones increased interleukin (IL)-10, tumor necrosis factor (TNF)-α, IL-6, and IL-8 cytokines in decidual stromal cells to defend against infection by *B. streptococcus* (9).

The development, activation, and survival of these cells depend on IL-5, which in the uterus is associated with the duration of the estrous cycle (5). In addition, the CCR3 receptor is another important molecule expressed in various cell types of the human endometrium and leukocytes such as eosinophils (10). The interaction of this receptor with its ligands, namely CCL11 (eotaxin 1), CCL24 (eotaxin 2), and CCL26 (eotaxin 3), triggers eosinophil recruitment (11 [Bibr B12]).

These studies have demonstrated eosinophil migration to tissues using different *in vivo* and *in vitro* models. However, the actual participation of the chemokine receptor CCR3 in this migration to the uterus as well as the formation of the edema that accompanies this event needs to be clarified. Therefore, studies about the mechanisms involved are necessary to understand the role of these cells in the reproductive physiology. Hence, we showed that the CCR3 receptor effectively participated in eosinophil migration to the uterus of ovariectomized mice and was important for the formation of edema under stimulation with 17β-estradiol (E2).

## Material and Methods

### Animals

All animal procedures were carried out in accordance with the standards of Guide for Care and Use of Laboratory Animals (National Institutes of Health) and were approved by the Ethics Committee on the Use of Animals of Federal University of Sergipe (UFS) under protocol number 38/2014.

Female C57BL/6 mice (20–25 g, n=5–8 per group) provided by the Animal House of UFS were kept under standard housing conditions. During the experiments, animals were randomly distributed among three experimental groups.

### Ovariectomy

The mice underwent surgical bilateral ovariectomy through a dorsal incision under anesthesia with 100 mg/kg of ketamine and 10 mg/kg of xylazine (Syntec^®^, Brazil). Additional anesthetic doses were given throughout the procedure as needed to maintain a constant level of anesthesia, determined by pupil constriction and absence of reflexive withdrawal of the hind limbs, indicative of adequate anesthesia. After removal of the ovaries, the dorsal wall was sutured and the cutaneous incisions closed with 10-mm calipers. As prophylaxis, animals received the anti-inflammatory flunixin meglumine (EquiMed Staff^®^, USA) at a dose of 2 mg/kg prior to surgery (*im*). The experimental procedures started 15 days after surgery.

### Time and dose of E2 to induce uterine edema

The dose and time for estrogen to induce uterine edema was determined with a single subcutaneous (*sc*) injection of E2 (Merck^®^, Germany; 100 μg/kg in inguinal region), and 6, 12, and 24 h after the injection the animals were anesthetized with ketamine (100 mg/kg) and xylazine (10 mg/kg), and euthanized by cervical displacement. The control group received only the vehicle sesame oil (SO; volume of 50 μL; Merck^®^). The uteruses were removed and weighed. Subsequently, the same procedure of E2 injection was followed using doses of 0.1, 1, 10, and 100 μg/kg, and organ collection was performed at the optimal time indicated in the previous experiment.

### Air pouch model

The effective dose of the CCR3 antagonist that reduced eosinophil migration was determined using the air pouch model, applied on anesthetized mice. These animals were depilated in the middle dorsal region, and 2.5 mL of sterile air was injected via *sc* on day 0, while on day 3 the same volume of the sterile air was injected into air pouch. Eosinophil migration was induced on the sixth day, using recombinant mouse CCL11 (R&D System^®^, USA), reconstituted in 100 μg/mL of phosphate buffer saline (PBS, pH 7.2) containing 1% bovine serum albumin (BSA). CCL11 (0.08 pmol/kg) was injected into the air pouch and the exudate was collected 4 h after injection. The control group received an injection of 100 μL of sterile PBS with 1% BSA per animal.

Next, we investigated the dose of the CCR3 receptor antagonist SB 328437 (Tocris^®^, United Kingdom) required to block eosinophil migration into the air pouch in response to CCL11. SB 328437 was dissolved in PBS and Tween 80 (0.1%) and doses of 1, 3, and 10 mg/kg were injected intraperitoneally (*ip*), 30 min before the CCL11 injection (0.08 pmol/kg) into the air pouch. One experimental group received only CCL11 or PBS with Tween 80 (negative control). Four hours after drug injection, animals were anesthetized and euthanized by cervical displacement. The air pouch was washed with 1.5 mL of PBS/EDTA and the lavage was collected for total and differential cell counting.

Total leukocyte number was determined in a Neubauer chamber, diluted in the Turk’s solution. The differential leukocyte count was performed on slides of the washed samples stained with hematoxylin-eosin for characterization of at least 100 cells according to the normal morphological criteria.

### Effect of CCR3 antagonist administration on eosinophil migration to the uterus

To investigate the effect of the CCR3 antagonism on eosinophil migration to the uterus, a group of mice received SB 328437 (*ip*) and 30 min later 100 µg/kg of E2 was administered. Another group received the vehicle 30 min before E2 administration. Twenty-four hours after E2 or SO administration, the uterus was collected, cleaned, weighed, and photographed.

Evaluation of eosinophil migration was estimated by measuring eosinophil peroxidase activity and counting eosinophils in tissue sections stained with orcein, as described below.

### Eosinophil peroxidase (EPO) assay

Eosinophil migration to the uterus was analyzed by measuring the activity of EPO using O-phenylenediamine (OPD; Merck^®^) as the substrate. Uterus samples (15 to 20 mg) were cut and held under ice before homogenization under ice in a solution (10 mmol/L, pH 6.0) containing PBS with 0.005% Tween 20 (1 mL/100 mg of tissue). The homogenate was centrifuged at 440 *g* for 15 min at 4°C. The supernatant was discarded and the pellet was resuspended with the same solution and centrifuged under the same conditions. Subsequently, the pellet was resuspended in PBS solution and hexadecyltrimethylammonium bromide (HTAB, 0.5%) and further centrifuged at 3000 *g* for 15 min at 4°C.

The supernatant was collected and 75 μL aliquots were mixed with 150 μL of substrate prepared with Tris-HCl (0.05 mol/L, pH 8.0), OPD (1.5 mol/L), and H_2_O_2_ (6.6 mmol). After 30 min of incubation at room temperature, the reaction was stopped with 75 μL of H_2_SO_4_ (1 mol/L) and the absorbance was determined at 492 nm with an ELISA Reader (Synergy MX^®^, BioTek, USA). In all analyses, PBS and HTAB were used together with the substrate containing OPD as a blank reaction. The results were normalized against the uterus weight used in the analysis and are reported in relation to 100 mg of tissue. A control experiment was carried out to ensure the linearity of the detection method of EPO activity with the dilutions 1/2, 1/10, and 1/100 (data not shown).

### Histological analysis

The uterus was immersed in 4% paraformaldehyde (for 18 h) for histological analysis. These were cut into 3 or 4 pieces and immersed in 70% alcohol and then embedded in paraffin. Tissues were sliced (5-µm sections) and stained with orcein (dye solution prepared with orcein, KCN, concentrated HCl, saturated urea, and 70% alcohol), specific for eosinophils (12). The slides were blind and morphometry was examined under a microscope with 100× magnification. All eosinophils present in the stromal region were counted (to increase the representativeness of the count; 3 sections were counted per slide). Data are reported as the mean of 10 fields.

### Statistical analysis

Data are reported as means±SE. Statistical calculations were performed with GraphPad Prism 7.0 (GraphPad Software, USA). The specific statistical tests as well as the number of animals (n) and experimental repetitions are indicated in the respective legends of the figures. A P value <0.05 was considered statistically significant.

## Results

### Effect of E2 administration on edema and EPO activity in the uterus

**Figure 1:**
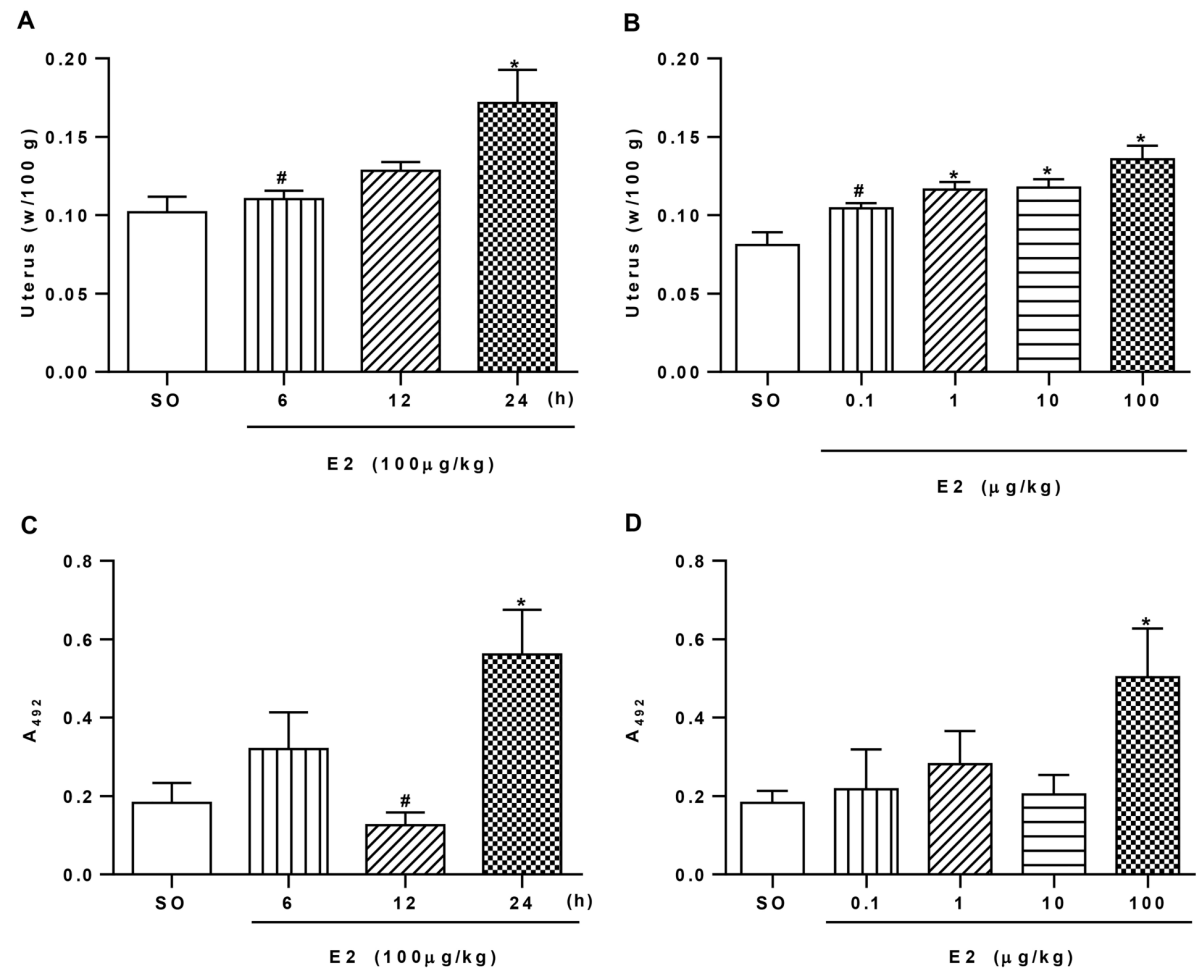
Time and dose of 17-β-estradiol (E2) that promoted edema and eosinophil migration to the uterus. **A**, The uterus of animals injected with E2 (100 μg/kg, *sc*) was collected and weighed (w) 6, 12, and 24 h after injection. The sesame oil group (SO, *sc*) was used as control. **B**, E2 doses of 0.1, 1, 10, and 100 µg/kg (n=10/group) were given and the uterus was collected and weighed 24 h later. **C** and **D**, The uteruses of the two experiments were used for the eosinophil peroxidase assay, reported as absorbance (A_492_). Data are reported as means±SE (3 independent experiments; n=5). *P<0.05 compared with SO group. ^#^P<0.05 compared with E2 dose in 24 h (one-way ANOVA with Tukey *post hoc* correction).

Analysis of the uterus wet weight demonstrated that after 24 h, the E2-injected animals (100 μg/kg) presented significant uterine edema, causing an increase of 40% in uterus weight compared with the group that received only SO (Figure 1A). Furthermore, different doses of E2 (0.1, 1, 10, and 100 μg/kg) were tested and doses of 1, 10, and 100 μg/kg were effective in inducing uterine edema 24 h after E2 administration. Nevertheless, the E2 dose of 100 μg/kg was the most effective, increasing organ weight by 40% (Figure 1B).

Thereafter, we evaluated the effect of E2 on the activity of the EPO enzyme. First, we observed that the 6-h interval promoted an increase of 43% in EPO activity, but the difference was not statistically significant. Thus, 24 h later, EPO activity increased 67% after E2 administration compared to SO (Figure 1C). Among the E2 doses tested (0.1-100 μg/kg), only 100 μg/kg increased (64%) EPO activity (Figure 1D).

### CCR3 antagonist (SB 328437) inhibited eosinophil migration to the uterus

**Figure 2:**
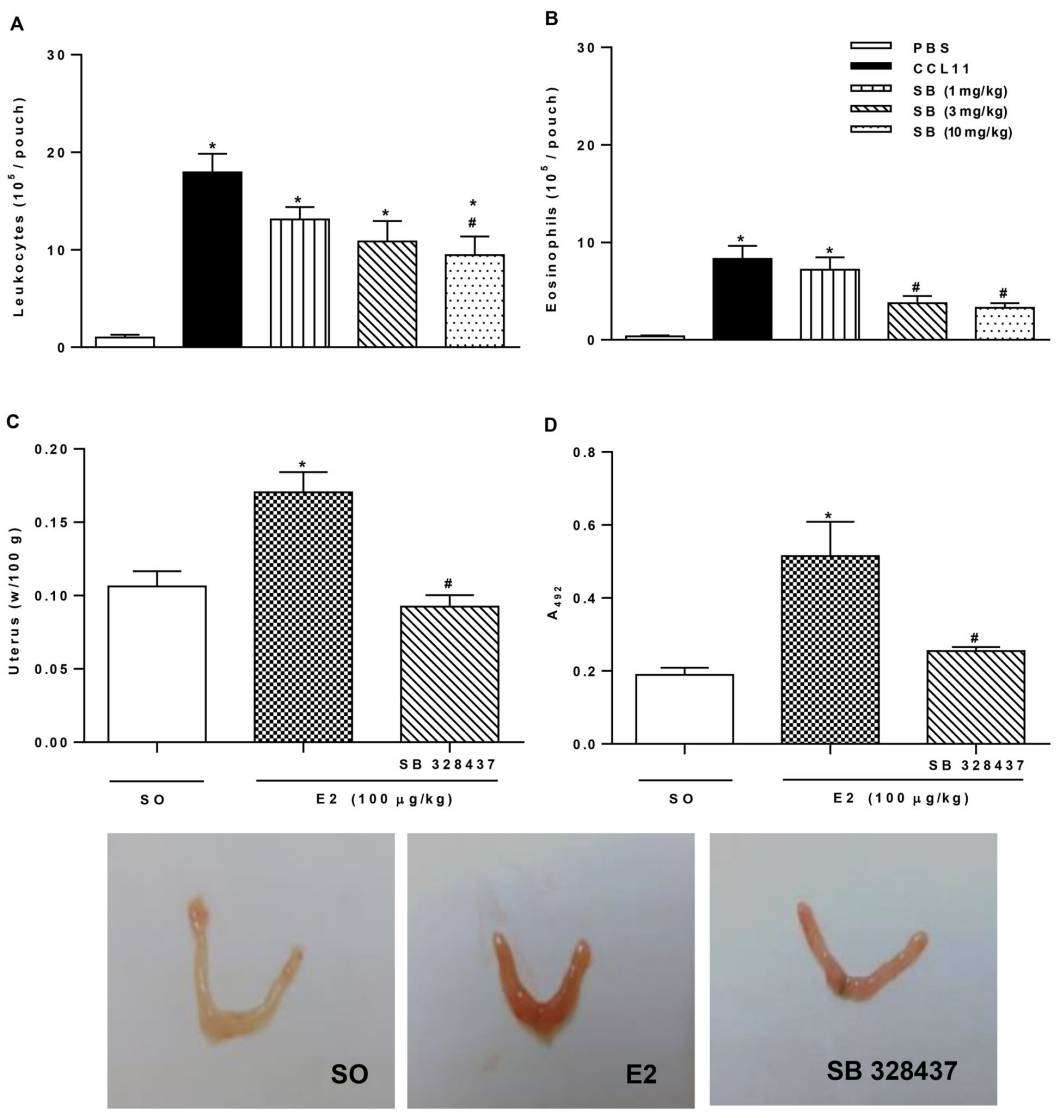
CCR3 antagonist dose that inhibited eosinophil migration. CCL11 (0.08 pmol/kg) was administered (*sc*) into the air pouch, and 4 h later approximately 1 mL of the exudate was collected for evaluation of (**A**) total leukocytes and (**B**) eosinophils. The antagonist dose (SB 328437; 3.0 mg/kg) was administered to the mice 30 min before 17-β-estradiol (E2) administration (100 µg/kg), and 24 h later the uterus was weighed for evaluation of (**C**) edema and (**D**) eosinophil peroxidase assay. Data are reported as means±SE of 2 independent experiments. *P<0.05 compared to PBS group (n=6) or SO group (n=5) and ^#^P<0.05 compared to CCL11 group (n=8) or E2 (n=5) (one-way ANOVA with Tukey *post hoc* correction). The photographs show the uterine atrophy/hypertrophy of ovariectomized mice treated with SO, E2, or with SB 328437 and E2.

Initially, the air pouch model was used to determine the CCR3 antagonist dose capable of inhibiting eosinophil migration in response to CCL11 (0.08 pmol/kg). CCL11 administration increased the total leukocyte and eosinophil migration into the air pouch (Figure 2A and B). In contrast, total leukocyte migration decreased in animals treated with CCR3 antagonist only at the dose of 10 mg/kg (Figure 2A). However, eosinophil migration was inhibited at the doses of 3 and 10 mg/kg (Figure 2B). Therefore, the lowest dose inhibiting eosinophil migration to the air pouch was used for the further experiments.

As shown in Figure 2C, the CCR3 antagonist inhibited uterine weight gain by 46% compared to the E2-injected group. Macroscopic evaluation of uterus tissue (Figure 2) demonstrated edema (turgid appearance) and uterine hyperemia following E2 administration compared to the SO group. These effects were greatly lower in mice treated with CCR3 antagonist. Corroborating the result, the increase in EPO activity was also inhibited (50%) by CCR3 antagonist compared to the E2-injected group (Figure 2D).

**Figure 3:**
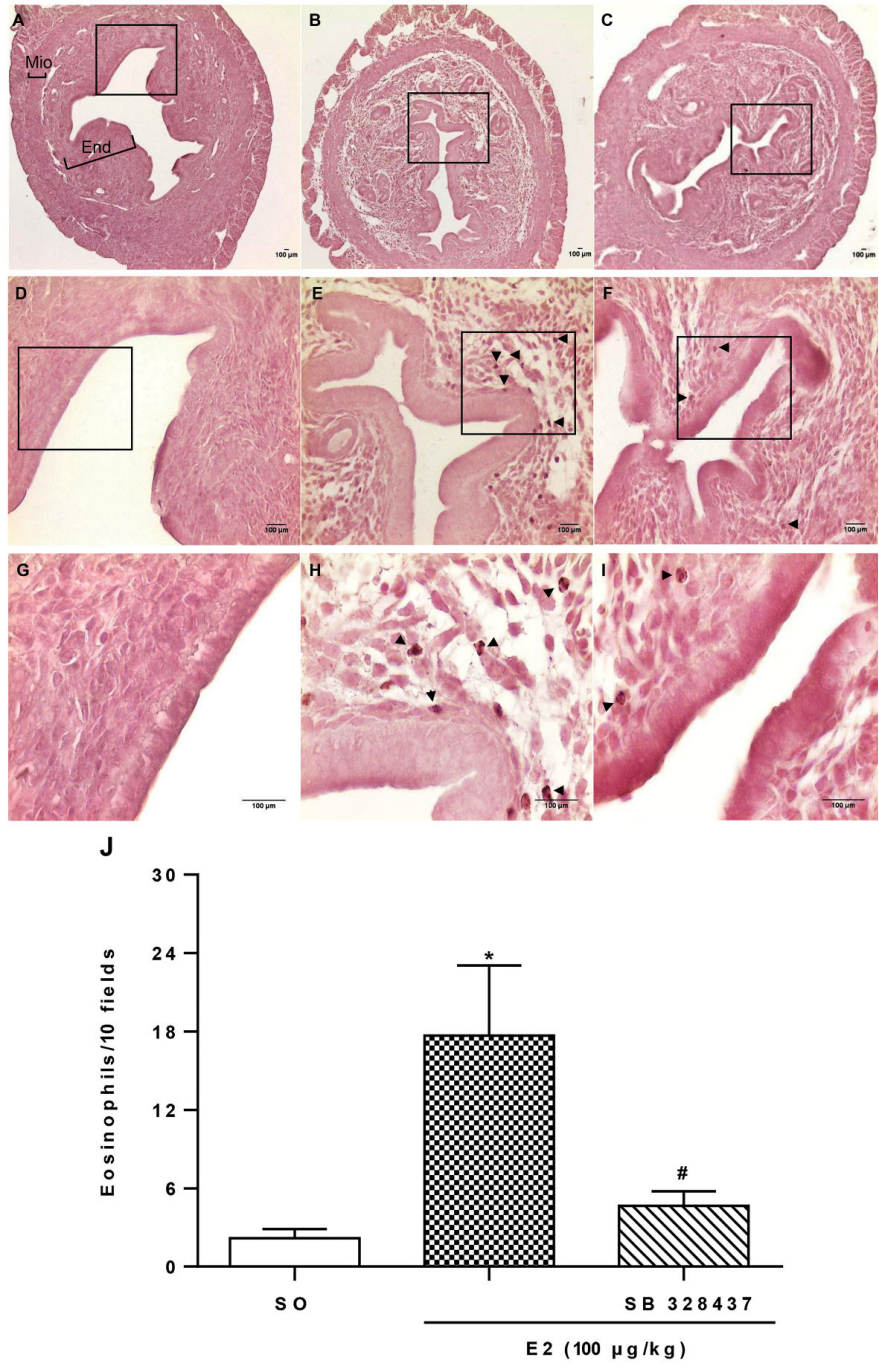
Effect of CCR3 antagonism on 17-β-estradiol (E2)-influenced eosinophil migration and uterine edema. Cross-sections of uterine tissue (5 μm) of ovariectomized C57BL/6 mice stained with orcein. The animals were injected with sesame oil (SO) (**A**, **D**, **G**), E2 (100 μg/kg; **B**, **E**, **H**), or E2 and SB 328437 (3 mg/kg; **C**, **F**, **I**) and 24 h later the uteruses were collected (n=5). In panel **A**, the black bars show the uterine regions of the myometrium (Mio), and endometrium (End). The regions represented by **A**–**C**, **D**–**F** or **G**–**I** are photographs at 10, 40, and 100× magnification, respectively; scale bars: 100 μm. The arrows indicate eosinophils stained with orcein. **J**, Eosinophil quantification as the average count of 10 fields (n=5). Data are reported as means±SE. *P<0.05 compared to SO and ^#^P<0.05 compared to E2 alone (one-way ANOVA with Tukey *post hoc* correction).

Moreover, to confirm the inhibition of eosinophil migration after CCR3 antagonist administration, morphometry of uterine sections stained with an alkaline solution of orcein was performed (Figure 3A–I). The eosinophil count in the uterus was 87% greater compared to the SO group (Figure 3J) after E2 administration, clearly seen in Figure 3E and H. In contrast, panels F and I demonstrate remarkable inhibition of E2-induced eosinophil migration by the pretreatment with CCR3 antagonist, reducing the eosinophil number by 73% (Figure 3J).

## Discussion

The findings of our study demonstrated that the CCR3 selective antagonist (13) inhibited eosinophil migration into the uterus as well as uterine edema of ovariectomized mice in response to E2.

It is known that eosinophils constitutively express the CCR3 receptor in mice, and the interaction with their several ligands promotes recruitment of these cells by tissues. Lee et al. (14) found a uterine eosinophil chemotactic factor (ECF-U) regulated by estrogen in immature rats, and concluded that the estradiol-stimulated increase in uterine eosinophils is due to the influence of ECF-U. Subsequently, it was established that IL-5, another important cytokine for tissue eosinophilia, is not necessary for eosinophil recruitment to the uterus (4). This supported the hypothesis that other pathways are involved in this process. Gouon-Evans and Pollard (5) demonstrated that CCL11-deficient mice did not contain eosinophils in the uterus and that this chemokine could be the ECF-U factor dependent on E2.

It is known that CCR3 is a rather promiscuous receptor, which can be activated by several ligands. Thus, we suggest that estrogen might have been stimulating the production and/or release of CCR3 ligands by uterus resident cells. In the uterus, such ligands, namely CCL5, CCL2, CCL3, and CCL7, are expressed and their production/release can be influenced by E2 (15). Moggs et al. (16) evaluated the expression of genes related to the E2-induced uterotrophic process and observed activation of the genes for CCL2, CCL7, and CCL11 between 4 and 72 h after induction. Concomitant to this, it has been established that epithelial, luminal, and glandular cells of the human endometrium express the CCR3 receptor and CCL11 and that the stimulation by estrogen promotes CCL11 release (10).

Additionally, the literature shows conflicting results regarding CCR3/CCL11 in asthma. A previous study with CCR3-deficient mice found that the chemokine did not affect the amount of eosinophils in the lungs of asthmatic mice (17). However, the importance of CCR3/CCL11 to eosinophil migration in asthmatic lungs is a target for drug investigation (18).

Moreover, other cells may influence the amount of eosinophils in the uterus in response to E2 through the release of mediators important for the survival of eosinophils. Recently, it was found that the innate lymphoid type 2 cells (ILC2), which release IL-5, are present in the uterus in an estrogen-dependent manner (19). Thus, we suggest that these cells can control the amount of eosinophils in the uterus.

Our results demonstrated for the first time that the CCR3 receptor may be involved in the formation of uterine edema. However, Gouon-Evans and Pollard (5) showed that the increase in uterine weight was not influenced by the quantity of eosinophils or CCL11 in the tissue.

The exact role of edema in cervical remodeling is not completely understood. Nonetheless, it is known that uterine edema in the cervix occurs via induction of vascular endothelial growth factor (VEGF) to permit leukocyte passage, at the same time remodeling and preparing itself for the final fetal passage (20). Considering the findings of our study on eosinophil migration and uterine edema, it is possible to state that the CCL11-CCR3 pathway is important for these phenomena. Despite this, the possibility that other CCR3 ligands act in this process should not be excluded.

The relationship of this receptor with the formation of uterine edema is still poorly understood and needs to be better explored. Nevertheless, our findings help elucidate the mechanism of eosinophil migration and edema formation and contribute to future studies investigating the role of eosinophils in uterine physiology.

## References

[1] Bjersing L, Borglin NE (1964). Effect of hormones on incidence of uterine esosinophilia in rats. Acta Pathol Microbiol Scand.

[2] Tchernitchin A (1972). Radioautographic study on the effect of estradiol-17, estrone, estriol, progesterone, testosterone and corticosterone on the in vitro uptake on 2,4,6,7-3 H estradiol-17 by uterine eosinophils of the rat. Steroids.

[3] Tchernitchin NN, Clavero A, Mena MA, Unda C, Villagra R, Cumsille M (2003). Effect of chronic exposure to lead on estrogen action in the prepubertal rat uterus. Environ Toxicol.

[4] Robertson SA, Mau VJ, Young IG, Matthaei KI (2000). Uterine eosinophils and reproductive performance in interleukin 5-deficient mice. J Reprod Fertil.

[5] Gouon-Evans V, Pollard JW (2001). Eotaxin is required for eosinophil homing into the stroma of the pubertal and cycling uterus. Endocrinology.

[6] Shamri R, Xenakis JJ, Spencer LA (2011). Eosinophils in innate immunity: an evolving story. Cell Tissue Res.

[7] Vicetti Miguel RD, Quispe Calla NE, Dixon D, Foster RA, Gambotto A, Pavelko SD (2017). IL-4-secreting eosinophils promote endometrial stromal cell proliferation and prevent Chlamydia induced upper genital tract damage. Proc Natl Acad Sci USA.

[8] Kovats S (2015). Estrogen receptors regulate innate immune cells and signaling pathways. Cell Immunol.

[9] Castro-Leyva V, Zaga-Clavellina V, Espejel-Nuãez A, Vega-Sanchez R, Flores-Pliego A, Reyes-Muãoz E (2017). Decidualization mediated by steroid hormones modulates the innate immunity in response to group b streptococcal infection in vitro. Gynecol Obstet Invest.

[10] Zhang J, Lathbury LJ, Salamonsen LA (2000). Expression of the chemokine eotaxin and its receptor, CCR3, in human endometrium. Biol Reprod.

[11] Provost V, Larose MC, Langlois A, Rola-Pleszczynski M, Flamand N, Laviolette M (2013). CCL26/eotaxin-3 is more effective to induce the migration of eosinophils of asthmatics than CCL11/eotaxin-1 and CCL24/eotaxin-2. J Leukoc Biol.

[B12] Goldstein DJ (1963). Selective staining of eosinophil granules in sections by alkaline orcein in a concentrated urea solution. Stain Technol.

[13] White JR, Lee JM, Dede K, Imburgia CS, Jurewicz AJ, Chan G (2000). Identification of potent, selective non-peptide cc chemokine receptor-3 antagonist that inhibits eotaxin-, eotaxin-2-, and monocyte chemotactic protein-4-induced eosinophil migration. J Biol Chem.

[14] Lee YH, Howe RS, Sha SJ, Teuscher C, Sheehan DM, Lyttle CR (1989). Estrogen regulation of an eosinophil chemotactic factor in the immature rat uterus. Endocrinology.

[15] Robertson SA, Allanson M, Mau VJ (1998). Molecular regulation of uterine leukocyte recruitment during early pregnancy in the mouse. Placenta.

[16] Moggs JG, Tinwell H, Spurway T, Chang HS, Pate I, Lim FL (2004). Phenotypic anchoring of gene expression changes during estrogen- induced uterine growth. Environ Health Perspect.

[17] Humbles AA, Lu B, Friend DS, Okinaga S, Lora J, Al-garawi A (2002). The murine CCR3 receptor regulates both the role of eosinophils and mast cells in allergen-induced airway inflammation and hyperresponsiveness. Proc Natl Acad Sci USA.

[18] Chen Y, Zhang Y, Xu M, Luan J, Piao S, Chi S (2017). Catalpol alleviates ovalbumin-induced asthma in mice: Reduced eosinophil infiltration in the lung. Int Immunopharmacol.

[19] Bartemes K, Chen CC, Iijima K, Drake L, Kita H (2018). IL-33-responsive group 2 Innate Lymphoid Cells are regulated by female sex hormones in the uterus. J Immunol.

[20] Donnelly SM, Nguyen BT, Rhyne S, Estes J, Jesmin S, Mowa CN (2013). Vascular endothelial growth factor induces growth of uterine cervix and immune cell recruitment in mice. J Endocrinol.

